# Supplementary material for: Swarm: A federated cloud framework for large-scale variant analysis
Source: PLoS Comput Biol. 2021 May 12;17(5):e1008977. doi: 10.1371/journal.pcbi.1008977 (PMC8143397; doi:10.1371/journal.pcbi.1008977)
Supplement: Attachment — Submitted filename: Swarm - Response to Reviewers.pdf [file pcbi.1008977.s001.pdf]

We thank both reviewers for their constructive and thoughtful comments, which have helped substantially improve our manuscript. The following are our point-to-point responses to each reviewer's questions.

Review #1

1. The variation in the execution time was not shown in Figures 2, 3, or 4. Although the variations may not be substantial since the pipeline under investigation is purely computational, as the authors stated on page 7, the information on the execution time variation can help determine whether one setting is statistically better than another. The authors could conduct significance tests to compare the differences across groups as well.

> Great feedback - we now plotted standard deviation in the figures providing information about runtime, and added P-values to the experiments and indicated them in the figures when applicable.

2. It was unclear how much time is needed to set up the Swarm environment proposed by the authors. The computational overhead of setting up the platform does not seem to be included in the current analyses. The authors could package the setup codes in a single executable or via docker to expedite and simplify the setup process.

> Thanks for the feedback - We created a dockerized version of the tool (Github link: https://github.com/StanfordBioinformatics/swarm/blob/master/Dockerfile)

3. Table 3 compares the average running time for querying an example single nucleotide polymorphism (SNP) using Apache Presto. The precision of the execution time is somewhat limited in the current table. The authors could consider using `time` or other related Unix command to get the exact running time of the query using different numbers of compute nodes. Metrics of variations in different runs will be helpful here as well.

> Thanks for the feedback - we reran the experiment and reported the runtimes with higher accuracy in Table 3.

4. The authors could discuss how their proposed framework could accommodate federated machine learning tasks. More and more users are developing machine learning approaches to aggregate the contributions of different genetic variants in relation to their outcomes of interest (such as diseases, phenotypes, or other endpoints). It would be interesting to see if the proposed system not only provides simple summary statistics or results from data queries but also enables the transfer of gradients or any other intermediates required for

federated learning. This could greatly enhance the potential impact of the proposed cloud computing framework.

Excellent feedback - we added a new feature in Swarm for handling *ad hoc* computation. As a proof of concept, we also implemented a version of Swarm that supports a federated learning use case. Users can provide an ad-hoc Docker image and execute a task on one platform (e.g., training a model), and move the trained model to a second platform. Swarm executes the first task on the first platform, transfers the output model/files to the other platform, and continues the computation by creating a new container on the second platform. For this proof of concept, we selected a basic polygenic risk score (PRS) analysis using PLINK, an open-source whole genome association analysis toolset.

5. Figure 4 showed that the non-PVM (non-preemptible) environment and the N-2 PVMs (preemptible) + 2 non-PVMs setups have similar average execution time, while the monthly cost of the non-PVM environment is higher, since non-PVM generally cost more. Did the authors experience any preemption when running the experiments with PVM? If so, how does that affect the computation time and cost? What are the methods implemented in Swarm to enable a fast resumption of the unfinished computation?

Thank you for the feedback. Currently, we do not have any self-healing mechanism in Swarm; one simple approach is to check if the output files exist already and avoid recomputation, but tracking potential corrupted files would be a major challenge here. Google BigQuery, AWS Athena and Apache Presto have different approaches for handling resilience within themselves, such as automatic retries to deal with minor storage and network availability downtime; more info around fault Tolerance:

Apache Presto: Sethi, Raghav, et al. "Presto: Sql on everything." *2019 IEEE 35th International Conference on Data Engineering (ICDE)*. IEEE, 2019.

Google BigQuery: “*In the event of a machine-level failure, BigQuery will continue running with no more than a few milliseconds delay. All queries should still succeed. In the event of a zonal failure, no data loss is expected. Soft zonal failure, such as resulting from a power outage, destroyed transformer, or network partition, is a well-tested path.*”
https://cloud.google.com/bigquery/docs/availability

Amazon Athena (durability of 99.999999999%):
https://docs.aws.amazon.com/athena/latest/ug/security-resilience.html

6. The authors specified the compute nodes used for the third experiment (n1-standard-4 on Google Cloud Platform). However, the computing environment for other experiments was not specified. Different computational environments likely have different computational performance and cost.

> Thank you for your accurate feedback. We updated all the figures, and specified the type of the instances, except for experiments related to BigQuery and Amazon Athena as they are both serverless.

7. Readers may be interested in a quick comparison between the proposed framework and some alternatives. For example, how is the computation time and cost of Swarm compare with a naïve implementation of SQL database that requires moving all relevant data across the platforms?

> Great feedback - we added a new experiment using MySQL RDBMS (Figure 5 (a)). For this experiment, we loaded the entire 1000 Genomes dataset without the genotyping columns. The MySQL queries were executed on different *n1-standard* machine types on Google Cloud Platform. This evaluates execution time, but egress of the raw data would be of similar cost to the serverless platforms assuming the MySQL instance is running in a commercial cloud environment.
>
> We also conducted a new experiment about the performance of Apache Presto importing CSV [a traditional row based storage format] vs. Parquet [a columnar storage format]. In order to compare the performance of Apache Presto on a CSV file and a Parquet file based on rsID search, the same 1000 Genomes dataset without genotyping information was utilized. Similar to the third experiment, the queries on Apache Presto were run using a different number of n1-standard machine type-based worker nodes on Google Cloud Platform.

Additional minor comments:

1. Page 7, paragraph 4: “preemtible" should be “preemptible”.

> Thank you - we fixed it.

2. For some reason, the GitHub Link provided in the manuscript does not work for me. I am not sure if it requires any specific permission setup (e.g., within the authors' research groups) to access the source codes.

Thanks for the feedback - We created a dockerized version of the tool and improved documentation around how to configure Swarm (Github link: https://github.com/StanfordBioinformatics/swarm)

Review #2

Bahmani et al. sescribe Swarm as a federated framework for variant analysis. Swarm offers to perform computational analyses on large genomics datasets hosted on different cloud platforms, enabling collaboration within or between different organizations and institutions, and facilitating

multi-cloud solutions. As such, the method is fairly generic and could accelerate discoveries in small teams and larger collaborative studies, including between research and healthcare. It could also reduce costs of scientific studies, given the data movement is expensive in the cloud world.

Frameworks for federated computation are likely to increase in importance, as are frameworks to

promote minimal data motion and facilitates crosstalk between datasets stored on different cloud platforms. This is an important contribution, and I overall liked this manuscript, although I have a couple of remaining questions:

The authors thank the reviewer for the accurate summary.

The authors applied swarm to GCP BigQuery, AWS Athena and Apache Presto on an Apache Hadoop cluster. How easy is it to port swarm to new clouds, including non-commercial clouds and clouds used outside of the USA for example?

Thank you for the suggestion. We provided a section in GitHub on how to extend Swarm for other platforms.

https://github.com/StanfordBioinformatics/swarm/blob/master/doc/Extending.md

Could the authors present cost estimates for some generic operations? Specifically, what compute costs can be saved by using swarm when integrating data from distinct cloud platforms?

Thank you for the great feedback - Three are at least five major factors in terms of costs: 1) Storage, 2) Compute, 3) Egress fee, 4) Costs associated with Ingress and 5) Maintenance costs; these factors are mainly associated with the underlying service models (IaaS or PaaS). Swarm as a SaaS application can work with both service models (BigQuery/Athena or Presto/MySQL or a combination of all). The major contribution in terms of cost saving is around egress fee, the potential storage cost (e.g., duplicate tables), and likely maintenance costs (e.g., reducing the cost of security and privacy features by not moving raw data).

We provided two examples for the egress cost saving (Line 150 and Line 167), and one example (Figure 4 (c)) around the monthly costs of preemptible vs. non-preemptible cluster computers for Apache Presto. The maintenance costs associated with this approach is much higher compared to the serverless solutions.

We also investigated and measured the advantage of partitioning and clustering schemes in BigQuery, Athena, and Presto, and found very large benefits to partitioning the large datasets in use. Another area of investigation was the difference between a row-based CSV format commonly used in data warehousing applications, and a columnar compressed Parquet format in Athena and Presto, which is similar to the internal Capacitor format used by BigQuery.

Additionally, the authors should discuss how anomalies are dealt with in the framework. In particular, is Swarm tolerant to issues/anomalies that may occur during cloud computing,

and which unintendedly can increase the costs of a study quite substantially – for example by delaying the pace of a large-scale project (Yakneen et al. Nat Biotechnol 2020). Have the authors considered self-healing for Swarm?

Thank you for the feedback. Currently, we do not have any self-healing mechanism in Swarm; one simple approach is to check if the output files exist already and avoid recomputation, but tracking potential corrupted files would be a major challenge here. Google BigQuery, AWS Athena and Apache Presto have different approaches for handling resilience within themselves, such as automatic retries to deal with minor storage and network availability downtime; more info around fault tolerance:

Apache Presto: Sethi, Raghav, et al. "Presto: Sql on everything." *2019 IEEE 35th International Conference on Data Engineering (ICDE)*. IEEE, 2019.

Google BigQuery: *"In the event of a machine-level failure, BigQuery will continue running with no more than a few milliseconds delay. All queries should still succeed. In the event of a zonal failure, no data loss is expected. Soft zonal failure, such as resulting from a power outage, destroyed transformer, or network partition, is a well-tested path."*
https://cloud.google.com/bigquery/docs/availability

Amazon Athena (durability of 99.999999999%):
https://docs.aws.amazon.com/athena/latest/ug/security-resilience.html

I did not see a software maintenance plan. Will the swarm framework be actively maintained? This may be very important. Cloud frameworks are short lived, and this is a rapidly moving field.

Thanks for your comment. We have improved our GitHub page by providing more description and we also created a dockerized version of Swarm. We plan to continue adding new features and supporting the tool.

Could swarm be used for functions beyond large-scale variant analysis?

Excellent question - we added a new feature in Swarm for handling ad hoc computation. As a proof of concept, we also implemented a version of Swarm that supports a federated learning use case. Users can provide an ad-hoc Docker image and execute a task on one platform (e.g., training a model), and move the trained

model to the second platform and utilize it. Swarm executes the first task on the first platform, transfers the output model/files to the other platform, and continues the computation by creating a new container on the second platform. To demonstrate Swarm's utility on ad hoc computation, we chose basic polygenic risk score (PRS) analysis using PLINK, an open-source whole genome association analysis toolset.

We also hope to investigate generic tabular queries and data federation by making the APIs more modular and not intrinsically specific to variant and annotation data.
